# Theme and variations: activation and regulation of gasdermin-mediated inflammation

**DOI:** 10.1042/BST20250185

**Published:** 2026-06-24

**Authors:** Eleanor Yeats Rothera, Soyar Horam, Yizeng Hu, Szu-An Lin, Aidan J. David, Tsan Sam Xiao

**Affiliations:** Department of Pathology, Case Western Reserve University, Cleveland, OH 44106, U.S.A.

**Keywords:** gasdermin, inflammation, membrane targeting, post translational modification, protease, pyroptosis

## Abstract

Gasdermins are effectors for pyroptosis, a highly inflammatory form of cell death. Mammalian gasdermin (GSDM) family members harbor N-terminal domains (NTDs) that bind membrane phospholipids and assemble oligomeric pores. Their C-terminal domains are regulatory modules, which suppress the cytolytic function of the NTDs under homeostatic conditions, and in several cases mediate the recruitment of proteases that cleave GSDMs following upstream signaling. The initial model for gasdermin activation was that upon protease processing their NTDs localize to the plasma membrane to assemble oligomeric pores and mediate pyroptosis. Emerging evidence suggests fascinating variations of this paradigm. For example, cleavage-independent pyroptotic activities have been reported for several family members that undergo post-translational modifications such as S-acylation, PARylation, oxidation, or phosphorylation. Furthermore, some gasdermins associate with membranes from organelles such as mitochondria, and often play non-pyroptotic roles in cellular physiology. In the present mini-review, we briefly summarize the molecular mechanisms governing the activation of different gasdermin family members, focusing on protease processing as the most well-studied mechanism. This is followed by discussion of two aspects of gasdermin biology. Namely, cleavage-independent pyroptotic activities and the localization of gasdermins at mitochondria and nucleus implicated in pyroptotic and non-pyroptotic functions. The diverse mechanisms of gasdermin activation and regulation in response to different upstream signaling pathways demonstrate the versatility of this conserved family of pore-forming proteins in various aspects of cellular physiology throughout evolution. The pleiotropic functions of gasdermins in inflammatory disorders, antimicrobial defense, antitumor immunity, neurodegenerative disorders etc., suggest fertile ground for exploration of therapeutic avenues.

## Introduction

Gasdermins are a protein family recognized as effectors for pyroptosis, a highly inflammatory form of cell death [1–8]. Six family members gasdermin A (GSDMA), GSDMB, GSDMC, GSDMD, GSDME, and GSDMF/Pejvakin (PJVK), share a conserved two-domain structure. Except for PJVK, the N-terminal domains (NTDs) from gasdermins bind to negatively charged phospholipids such as cardiolipin or phosphoinositol, and assemble oligomeric pores in the membrane. The C-terminal domains (CTDs) serve dual regulatory functions: they suppress the cytolytic function of the NTDs under homeostatic conditions; in both GSDMD and GSDMB, their CTDs mediate the recruitment of respective proteases upon their activation using exosite interfaces [9–11]. As such, the CTD may be more appropriately designated as a regulatory domain that serves crucial functions in both resting and activated states, rather than just an autoinhibitory domain as described in most literature. A flexible linker region connects the NTD and CTD, which can be cleaved by various proteases to facilitate the release of the NTD. The NTD can engage lipids, undergo dramatic conformational changes, and assemble large oligomeric pores that insert into various membranes [8,12–15].

**Figure 1:**
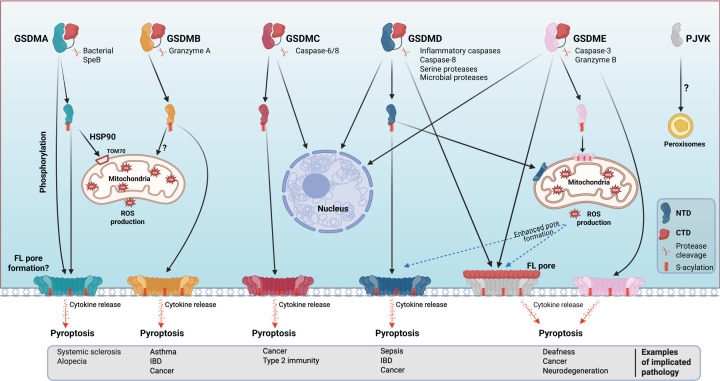
Activation and regulation of mammalian gasdermins Most gasdermin family members assemble pores at the plasma membrane to mediate pyroptosis upon activation through protease cleavage. Some of the proteases that activate each gasdermin are shown. Other post-translational modifications have been shown to activate gasdermins, in some cases without protease cleavage. Activated gasdermins have been reported to localize to intracellular membrane compartments besides plasma membrane, which may be achieved through a combination of intrinsic lipid affinity, post-translational modifications including S-acylation, utilization of chaperone proteins and other factors in the context of different cell types, tissues, and physiological or pathological conditions. The pleiotropic pyroptotic and non-pyroptotic functions of gasdermins in inflammatory disorders, antimicrobial defense, antitumor immunity, neurodegenerative disorders etc., suggest fertile ground for exploration of therapeutic avenues.

In the present mini-review, we briefly summarize the distinct molecular mechanisms governing the activation of different gasdermin family members, focusing on protease processing as the most well-studied mechanism in the context of selected physiological or pathological conditions. This is followed by discussion of two aspects of gasdermin biology that extends beyond the original paradigm of gasdermin function. Such ‘variations of the theme’, so to speak, include cleavage-independent pyroptotic activities following different post-translational modifications for several gasdermin family members. In addition, localization of gasdermins beyond plasma membrane, such as at mitochondria and nucleus, has revealed novel pyroptotic and non-pyroptotic functions (Figure 1). While the present mini-review only covers select aspects of gasdermin biology, other mechanisms of gasdermin activation and regulation through S-palmitoylation/acylation, oxidation, succination, phosphorylation, ubiquitination, or O-GlcNAcylation, etc. have been reported, and updated reviews have been published in the last year or so [15, 16, 17, 18, 19, 20, 21].

## Gasdermin D is regulated by host and microbial proteases

GSDMD is widely expressed throughout the body, particularly in epithelial cells and immune cells. As the most well-characterized gasdermin protein, human and mouse GSDMDs are cleaved by inflammatory caspases-1, 4, 5, and 11 downstream of the canonical and non-canonical inflammasome pathways [1–3]. Structural studies have revealed that activated inflammatory caspases bind GSDMD at an exosite distant from the tetrapeptide cleavage site (with a sequence of FLTD-275 in human GSDMD or LLSD-276 in murine GSDMD) at the GSDMD linker [9,10]. This mode of exosite binding involves anti-parallel beta sheets in caspases binding a hydrophobic pocket within the GSDMD–CTD, thus revealing a new role for the CTD as a caspase recruitment domain besides its function in autoinhibition. These findings were corroborated by a novel mass spectrometry technique called carbene footprinting, which confirmed the importance of the exosite in GSDMD recognition by inflammatory caspases [22].

Beyond inflammatory caspases, apoptotic caspase-8 was reported to process and activate GSDMD. During *Yersinia pestis* infection, inhibition of TAK1 activates caspase-8, which cleaves GSDMD to trigger pyroptosis in macrophages [23]. A similar mechanism was identified during influenza A infection, where caspase-8 activated GSDMD and amplified antiviral responses [24]. Neutrophil serine protease, elastase neutrophil expressed (ELANE), can cleave GSDMD to assemble pores that facilitate cytokine release [25,26]. In contrast to the above proteases that cleave GSDMD at its linker region and trigger pyroptosis, other proteases that cleave within its NTD have been shown to suppress GSDMD-mediated pyroptosis, such as apoptotic caspase-3/7 [23,27,28]. Several viral proteases target GSDMD to regulate pyroptosis, such as the enteroviral 3C protease that cleaves GSDMD at residue Asp193 [29], the SARS-CoV-2 3CL protease that cleaves at Gln29 and Gln193 [30,31], the African swine fever virus protease pS273R that cleaves at Gly107 [32] etc. These viral proteases thus function to suppress GSDMD-mediated cytokine release and/or pyroptosis, as a strategy to evade host antiviral innate immune responses.

## Gasdermin A activation by a bacterial protease

GSDMA is expressed in animals besides mammals, such as birds, sharks, amphibians, and reptiles, which suggests that GSDMA was present in the common ancestor of these animals [12]. Evolution analysis suggests that GSDMA is the predecessor of GSDMB, GSDMC, and GSDMD in the common ancestor of amphibians and mammals [33]. Notably, caspase-1 can cleave GSDMA from birds, reptiles, and amphibians at its linker region when co-expressed in HEK293T cells, which induces pyroptosis. This suggests the possibility that the expression and function of non-mammalian GSDMA are similar to those of mammalian GSDMD [33,34].

In mammals, GSDMA is mainly expressed in the upper gastrointestinal (GI) tract and skin, which is also the main infection area of bacteria such as *Streptococcus* [35]. Two studies revealed that human GSDMA can be cleaved at residue Gln246 by *Streptococcus pyogenes* exotoxin B (SpeB), which is a bacterial cysteine protease secreted by group A *Streptococcus* during skin infections [36,37]. Such cleavage produces an NTD fragment that can trigger inflammatory cell death, which may limit the spread of infections. Similar cleavage site is conserved in mouse GSDMA1 (mGSDMA1), although all mGSDMA isoforms 1–3 can be cleaved by SpeB presumably at different sites to induce pyroptosis. Because currently no endogenous mammalian protease has been reported to activate GSDMA, uncovering such proteases is expected to be a major focus of the field in the near future.

## Human gasdermin B is characterized by isoform diversity

GSDMB is predominantly found in the digestive and respiratory tracts. The functions of human GSDMB are more diverse compared with other members of the gasdermin family, and can vary depending on different isoforms and activation mechanisms [38,39]. Among the multiple isoforms of human GSDMB, two harbor exon 6, which contains a motif crucial for membrane insertion by the GSDMB–NTD and therefore are capable of mediating pyroptosis [40–42].

Similar to GSDMD, processing by different proteases may trigger or suppress GSDMB-mediated pyroptosis. For example, upon cleavage at residue Lys244 by a serine protease granzyme A, which is released by natural killer and cytotoxic T-cells, the exon 6-containing NTDs form pores to mediate pyroptotic death of tumor cells [11,43]. Such killing of tumors by lymphocytes potentiates antitumor immunity. Interestingly, structural studies revealed that a dimeric granzyme A engages two GSDMB molecules through an exosite interface at the GSDMB CTD [11], in a manner similar to the recognition of GSDMD CTD by inflammatory caspases [9,10]. Caspase 1 has been shown to cleave GSDMB at residue Asp236, which also results in GSDMB-mediated pyroptosis [44]. By contrast, cleavage by ELANE at GSDMB residue Met220 renders the resulting NTD non-pyroptotic, which may be a mechanism for suppressing GSDMB-mediated pyroptosis or temper cell death during antitumor immune response [42,45].

Independent of their function in pyroptosis, some of the GSDMB isoforms have been reported to regulate focal adhesion kinase, and promote epithelial maintenance and repair [46,47]. This is consistent with elevated GSDMB expression in patients with inflammatory bowel disease, which harbor GSDMB mutants that are implicated in defective epithelial restitution or repair. Clearly, GSDMB may function in pyroptosis or tissue repair depending on the expression of different isoforms in diverse tissues, likely downstream of distinct signaling pathways.

## Gasdermin C in antitumor immunity and type 2 immunity

GSDMC, originally identified in melanoma cells and named melanoma derived leucine zipper extra nuclear factor (MLZE), was associated with enhanced metastatic potential in melanoma cells [48]. Most GSDMC isoforms in mice are expressed in intestinal epithelial cells [49], and can be activated by diverse upstream stimuli. For example, under hypoxic stress, PD-L1 is translocated to the nucleus. This enhances expression of human GSDMC, which is cleaved by caspases-8 and -6 at residue Asp365 to mediate pyroptosis in breast cancer cells [50]. Paradoxically, such pyroptosis of cancer cells in the hypoxic region promoted tumor progression and correlated with poor prognosis, which was attributed to suppressed antitumor immunity in the tumor microenvironment (TME) [50,51]. Similarly, GSDMC expression and activation were elevated in colorectal cancer and pancreatic cancer tissues with poor prognosis, and murine GSDMC2/3/4 deficiency attenuated tumor progression in colorectal cancer models [52–55]. Recruitment of myeloid-derived suppressor cells into the TME under hypoxia and low-glucose conditions was proposed to account for such tumor-promoting effects mediated by GSDMC [55].

In contrast to the above, other studies suggested that GSDMC-mediated pyroptosis of cancer cells boosted antitumor immunity and sensitized cancer cells to treatment using poly (ADP-ribose) polymerase (PARP) inhibitors [56]. Here, GSDMC expression was elevated by IFNγ treatment, and GSDMC was cleaved in triple-negative breast cancer cells. The GSDMC-mediated cancer cell pyroptosis then augmented antitumor immunity by promoting the activation and tumor infiltration of cytotoxic and memory T cells [56]. Importantly, the effects of ectopic GSDMC expression in sensitizing cancer cells to PARP inhibitor treatment were observed in pancreatic cancer, colorectal cancer, liver cancer, and melanoma as well. Zhang et al. reported that tricarboxylic acid cycle metabolite α-ketoglutarate (α-KG) induced caspase-8/GSDMC-mediated pyroptosis, which also suppressed tumor growth and metastasis [57]. This α-KG-triggered caspase-8 cleavage occurred at GSDMC residue Asp240. It is clear that GSDMC from different species, cell types, treatment conditions, or stages of tumorigenesis may all impact the roles of GSDMC in antitumor immunity.

In addition to roles in antitumor immunity, GSDMC in intestinal epithelial cells also functions in type 2 immune responses against helminth infections [58,59]. Here, cathepsin S protease and mast-cell protease 1 from intraepithelial mast cells were shown to cleave GSDMC at multiple sites including Ala252, His263, Thr264, Phe284, Leu285, and Phe289. This did not lead to death of epithelial cells. Instead, it facilitated GSDMC-mediated release of a critical type 2 cytokine, interleukin-33, thus driving type 2 immunity [58]. A second mechanism for type 2 immunity is through goblet cell hyperplasia and regulation of lipid droplet accumulation or lipid synthesis by GSDMC. Here, GSDMC was localized to Rab7-positive vesicles such as late endosomes, through an unknown mechanism, to regulate lipid synthesis [59]. However, a separate study demonstrated that mice deficient in GSDMC1-4 showed no changes in epithelial homeostasis, and GSDMC was dispensable for inflammatory response and worm expulsion during infection by helminth such as *Nippostrongylus brasiliensis* [49]. Future work will need to address whether the use of different parasites and infection conditions contributed to the contrasting observations of GSDMC function in type 2 immunity.

## Gasdermin E is processed by caspase-3 and granzyme B

GSDME, also known as deafness, autosomal dominant 5 (DFNA5), was first implicated in autosomal dominant non-syndromic hearing loss in a Dutch family [60]. It was later discovered to be the most ancient member of the gasdermin family found in vertebrates and some invertebrates [12,34]. Human GSDME is cleaved by caspase-3 at residue Asp270, which leads to pyroptosis mediated by its NTD [61,62]. As caspase-3 is an executioner proteases in apoptosis, GSDME activation by caspase-3 switches the type of cell death from apoptosis to pyroptosis. It is therefore not surprising that various chemotherapeutic agents, such as cisplatin, etoposide, and raptinal, induce activation of caspase-3 and GSDME-mediated pyroptosis that enhances anti-tumor immunity [61–64]. Recent structural and biochemical studies suggest that the recognition of GSDME by caspase-3 is through direct cleavage of the DMPD-270 tetrapeptide within the interdomain linker [65], independent of the exosite interfaces observed for GSDMD or GSDMB. This study also revealed that S-palmitoylation/acylation of GSDME enhances its pore-forming activity, with Cys180 as the primary site of lipidation [65]. In addition to caspase-3, GSDME is cleaved by caspase-7 from non-primate mammals, however human caspase-7 has a different p10 subunit that renders it unable to cleave GSDME [66]. GSDME can also be activated by granzyme B from cytotoxic lymphocytes, which cleaves GSDME directly at Asp270 as well as activates caspase-3. The resulting pyroptosis enhances the phagocytosis of tumor cells, as well as the number and functions of tumor-infiltrating cytotoxic lymphocytes [63].

In addition to its role in antitumor immunity, GSDME has been reported to function in neuronal mitochondrial dysfunction that may contribute to neurodegeneration [67]. It is highly expressed in the neurons but not microglia, which express GSDMD. GSDME is activated by caspase-3-dependent cleavage upon treatment of neurons with mitochondrial toxins such as raptinal or rotenone that induce apoptosis, or upon overexpression of neurodegeneration-associated proteins such as TDP-43 [67]. The cleaved GSDME localizes to mitochondria in axons, and promotes mitochondrial depolarization, trafficking defects, and neurite retraction. In agreement, GSDME deficiency protected against neurite loss in amyotrophic lateral sclerosis (ALS) patient iPSC-derived motor neurons, as well as prolonged survival and reduced neuroinflammation in ALS mouse models [67]. Similarly, caspase-8 is significantly upregulated in tissues from patients with Alzheimer’s disease, which induces activation of caspase-3 and GSDME-dependent pyroptosis that exacerbates neuroinflammation [68]. As a result, the caspase-8–caspase-3–GSDME axis may be an important focus for studies of neurodegeneration disorders.

## Cleavage-independent activation of mammalian gasdermins in pyroptosis

The identification of mammalian gasdermin family as pyroptosis effectors originated from the seminal discovery that caspase cleavage of the two-domain protein GSDMD triggers the plasma membrane association and pore formation by its liberated NTD [1–8]. Since then, protease cleavage of mammalian gasdermins has been regarded as their primary activation mechanism in the field, in agreement with the conservation of protease processing for ancient gasdermins [69]. Interestingly, the identification of full-length or near full-length gasdermin proteins harboring gain-of-function mutations pre-dated their recognition as pyroptosis effectors. Two decades prior to the discovery of GSDMD, a gain-of-function mutation associated with hearing loss was identified in GSDME/DFNA5, which caused skipping of exon 8 resulting in premature termination of the open reading frame [60,70]. Expression of the mutant GSDME in yeast led to cell cycle arrest [71]. This suggested that a GSDME protein with a shortened C-terminal region may acquire a cytotoxic function that was relevant to hearing impairment. Similarly, autosomal dominant mutations in murine GSDMA3 (mGSDMA3) were discovered that caused hair loss and hyperkeratosis [72–75], which were later confirmed to endow the full-length mutant proteins spontaneous pyroptosis activities due to compromised NTD–CTD autoinhibition [1,4]. In fact, compromised autoinhibition through naturally occurring or designed mutations has been reported for almost all mammalian gasdermins, which led to the realization that mutant full-length gasdermins were capable of inducing pyroptosis at levels nearly comparable to the NTDs alone [4]. Subsequent structural studies of gasdermins further elucidated the mechanisms for autoinhibition, and demonstrated that mutations at or near the NTD–CTD interface led to cleavage-independent cytolysis independent of protease processing [76–78].

Besides mutations that disrupt autoinhibition, recent work suggested that full-length gasdermins are capable of inducing pyroptosis through other post-translational modifications. While investigating GSDMD S-palmitoylation/acylation, Du et al. reported that full-length human GSDMD was capable of inducing pyroptosis upon S-acylation at residue Cys191 [79]. Indeed, acylated and cleavage-deficient full-length GSDMD was shown to assemble 30-nm pore structures similar to the GSDMD NTD. A physiological scenario for this may be during oxidative stress, which was shown to elevate palmitoyltransferase expression and mitochondrial ROS production, thus promoting GSDMD acylation and membrane translocation in the full-length form. However, other contemporary studies suggested that full-length GSDMD was not able to bind lipids even after acylation, acylated full-length GSDMD did not induce significant pyroptosis [80–82], or acylation of GSDMD was not absolutely required for membrane association [83]. Clearly the roles of GSDMD lipid modification in modulating its pore-forming function in different cell types or under diverse stimulations merit further study. Again focusing on GSDMD Cys191, an intriguing mechanism for human GSDMD activation was reported through Cys191 modification by a small molecule GLP-1 receptor agonist, 6,7-dichloro-2-methylsulfonyl-3-N-tert-butylaminoquinoxaline (DMB) [84]. DMB acts through releasing autoinhibition as well as partially mimicking the hydrophobic S-acylation. The low levels of pyroptosis and toxicity upon DMB treatment suggests a novel therapeutic approach that takes advantage of such cleavage-independent GSDMD activation, for example in antitumor immunity. In addition, genetic analysis of *de novo* and inherited variants in an autism cohort identified a potential association between autism and the V41A mutation of GSDMD [79,85]. This mutation increased the acylation and concomitant cell death by full-length GSDMD, suggesting that the V41A mutation may promote GSDMD-mediated pyroptosis in autistic patients independent of protease processing. Collectively, these findings suggest that GSDMD S-acylation plays a significant role in cleavage-independent activation.

A different mechanism of post-translational modification was reported to activate full-length GSDME. Zhou and colleagues revealed that full-length GSDME could induce pyroptosis through a combination of two mechanisms from intense UV irradiation [86]. First, DNA damage from UV irradiation activated poly(ADP-ribose) polymerase 1 (PARP1), which promoted GSDME PARylation by a different enzyme PARP5 at linker residues Asp229/Glu233. This modification induced conformational ‘priming’ that released autoinhibition in GSDME. Secondly, UV irradiation promoted cytochrome C-catalyzed cardiolipin peroxidation and translocation to the mitochondrial outer membrane, which triggered the oxidative oligomerization of GSDME. This step was dependent on GSDME residues Cys156 and Cys180, although it is unclear whether these two Cys residues are involved in disulfide formation in GSMDE oligomers [87]. The concurrent stimulation of GSDME PARylation that releases autoinhibition and oxidative oligomerization synergistically promoted GSDME-mediated pyroptotic cell death and suppression of tumor growth. This was proposed to at least partially account for the anti-tumor effects of UV irradiation.

Phosphorylation is a common post-translational modification, and was reported to be an endogenous mechanism of GSDMA activation in the absence of cleavage [88]. Under conditions of severe starvation, the CTD of human GSDMA can be phosphorylated at Ser353 by an autophagy-initiating enzyme Unc-51-like autophagy-activating kinase 1 [88]. This disrupts autoinhibition and facilitates pore-formation by the full-length GSDMA. In agreement, mice harboring phospho-mimetic Ser to Asp mutation in mGSDMA exhibited skin inflammation and hyperplasia [88]. Collectively, emerging evidence suggests that various post-translational modifications of gasdermins may induce cleavage-independent activation of pyroptotic activities.

## Gasdermin localization at mitochondria

Gasdermins were initially thought to localize to the plasma membrane to assemble pores that lead to cell death [1–3]. It is now recognized that these lipid-binding proteins may indeed associate with other organelle membranes as well. Here, mitochondria and nucleus are two examples discussed below. Mammalian gasdermins harbor intrinsic affinity for cardiolipin [4,61,62,67,89–91], a phospholipid found almost exclusively in the inner mitochondrial membrane that contributes to mitochondrial quality control and bioenergetics [92]. For example, GSDMD–NTD was reported to fractionate with both plasma membrane and mitochondrial membrane [6,89]. This was correlated with studies using liposomes composed of either plasma membrane-like or mitochondria-like lipid bilayers that were bound equally well by GSDMD–NTD [5]. In addition, GSDMD–NTD bound to cardiolipin, localized at damaged mitochondrial membranes prior to plasma membrane damage [91]. This led to mitophagy, ROS production, release of mitochondrial proteins and DNA, and subsequent secretion of cytokines to the extracellular environment [91]. Such mitochondrial damage in turn promotes GSDMD cleavage and pore formation in a positive feedback loop that accelerates pyroptosis [91]. The physiological significance of such mitochondrial damage was borne out by reports showing that such damage led to cardiomyocyte dysfunction and myocardial inflammatory disorders [93–95]. In addition, the release of mitochondrial DNA was shown to activate the cGAS-STING [96] and AIM2 [91] signaling pathways, with the former reported to suppress endothelial regeneration during repair of lung injury [96].

Other gasdermins such as GSDMA and GSDME have also been reported to target mitochondria [67,90,97]. An early study of mGSDMA3 demonstrated that its NTD associated with heat shock protein 90, which was then delivered to the mitochondria by the importer receptor Tom70. Upon interacting with the mitochondrial chaperone Trap1, this complex then promoted mitochondrial ROS production [98]. Similarly, a gain-of-function Y344H mutant mGSDMA3 associated with mitochondria, diminish mitochondrial membrane potential, and induce ROS production [99]. Both studies suggested that mGSDMA3 may elevate inflammatory signaling through targeting mitochondria. In agreement, biochemical studies have shown that the NTD of GSDMA has a stronger affinity for cardiolipin at mitochondria than phosphoinositide at plasma membranes [4,100]. Kondolf and colleagues engineered a chimeric GSDMA/D construct that combined the NTD of GSDMA and the CTD of GSDMD plus a caspase-1-cleavable site in the linker [97]. When expressed in THP-1 cells, this chimera triggered early mitochondrial dysfunction, but delayed plasma membrane permeabilization and lysis. This echoes similar observations for GSDMD in localizing to mitochondria prior to plasma membrane, suggesting a shared mechanism of enhanced inflammatory signaling through mitochondria targeting by gasdermins prior to cell death [91].

GSDME has been reported to localize to the mitochondria in neurons [90], upon treatment with apoptosis-inducing reagents such as raptinal or rotenone that are characterized as mitochondrial toxins [67]. The rapid localization of GSDME to mitochondria drives mitochondrial damage and depolarization, release of ROS, and axon loss followed by cell death. Interestingly, expression of frontotemporal dementia or ALS-associated proteins TDP-43 and PR-50 induced GSDME-mediated mitochondrial damage and neurite loss. Importantly, GSDME deficiency preserved motor function and prolonged survival in a mouse model of ALS, and rescued neurite loss in a patient-derived ALS model. Since mitochondrial damage and neuronal death are hallmarks of neurologic inflammation and diseases, understanding the pathophysiological roles of GSDME and perhaps other gasdermins in mediating neuronal inflammation and cell death may suggest new strategies for targeting relevant neurodegenerative diseases.

Besides GSDMA, GSDMD, and GSDME, emerging evidence suggests that other gasdermins may also localize at mitochondria. For example, when the function of different fragments of GSDMB–NTDs were analyzed, those that included exon 6 and mediated pyroptosis localized to mitochondria and induce dysfunction, such as ROS production, mtDNA release, and loss of mitochondrial membrane potential [101]. Interestingly, a short version of GSDMB NTD harboring residues 1–220 without exon 6 was shown to localize to mitochondria but did not cause mitochondrial damage, suggesting that such short NTD without exon 6 may not assemble large pores [101]. Future studies will need to address the mechanisms for membrane association without pore assembly, as well as whether gasdermins assemble different pores at the mitochondria and plasma membrane.

## Gasdermin function at the nucleus

When colorectal cancer cells were subjected to stress conditions such as hypoxia or chemotherapy, GSDMD was shown to translocate to the nucleus [102]. This was based on immunohistochemistry images, but other approaches such as high-resolution microscopy or subcellular fractionation were not reported. This nuclear location facilitated GSDMD interaction with PARP-1 to inhibit its DNA–repair function, thus promoting apoptosis independent of pyroptosis. While this work revealed GSDMD function in the nucleus without cleavage, another study suggested that caspase-11-mediated cleavage of GSDMD was required for nuclear membrane permeabilization in neutrophils, leading to the extrusion of neutrophil extracellular traps as a defense mechanism against bacterial infection [103]. GSDMD was previously reported to be cleaved by caspase-3/7 within its NTD to generate an ∼10 kD fragment [23,27,28,104]. Interestingly, this cleavage of GSDMD was observed in response to certain dietary antigens [105]. The caspase-3/7-cleaved GSDMD fragment translocated to the nucleus in intestinal epithelial cells, and induced expression of CIITA and MHCII molecules that are important for tolerance of food antigens [105]. The above studies suggest that different fragments of GSDMD may harbor diverse nucleus-associated functions.

Similar to GSDMD, Wu and colleagues revealed a nuclear function of GSDMC independent of pyroptosis, based on the observation of elevated GSDMC expression during metastasis of pancreatic ductal adenocarcinoma (PDAC) and associated poor survival [54]. GSDMC was cleaved by ADAM17, and the resulting C-terminal fragment translocated to the nucleus. Of note, fractionation experiments in the same study also revealed the presence of GSDMC in mitochondria, ER, and Golgi besides nucleus. The nuclear GSDMC was shown to regulate expression of genes implicated in stemness, metastasis, and immune evasion, suggesting a tumor-promoting role of GSDMC. Pharmacological inhibition of GSDMC cleavage or prevention of its nuclear translocation suppressed GSDMC’s downstream targets and inhibited PDAC progression. These findings thus suggested GSDMC and ADAM17 as potential therapeutic targets for PDAC treatment.

Upon analyzing GSDME expression in psoriatic lesional skin, it was discovered that GSDME expression levels correlate with both psoriasis severity and response to biologics treatments [106]. This was at least partially attributed to the observation that GSDME associated with and regulated the nuclear translocation of p65 or c-jun to impact the expression of inflammatory mediators such as cytokines (IL1β), chemokines and S100A8/A9 etc from keratinocytes. This suggested a pyroptosis-independent function of GSDME in promoting nuclear translocation of transcription factors. Even though the majority of GSDME was located in the cytoplasm, minor fractions of the GSDME full-length protein was detected in the nuclear fraction, which was elevated upon treatment of keratinocytes with inflammatory cytokines. The mechanisms of how GSDME may regulate transcription factor localization to the nucleus remain to be explored.

It should be noted that our understanding of nuclear translocation and function of gasdermins remain incomplete, and in many cases preliminary. This is particularly true regarding the specific mechanisms for nuclear translocation of gasdermins or their cleavage fragments. Most reports relied on correlation from fractionation or fluorescence co-localization studies without characterizing how each gasdermin is specifically transported and regulated, how the expression of specific genes are regulated by each gasdermin, as well as how gasdermin functions may be cross-regulated by pyroptosis activities in the cytosol. Nonetheless, it is anticipated that these current limitations may stimulate active investigation to clarify the underlying mechanisms.

## Conclusion

More than a decade after the discovery of mammalian gasdermins as effectors for pyroptosis, significant insights have been gained regarding the activation and regulation of gasdermins through protease processing and other post-translational modifications. A balance between activation and inhibition of gasdermins allows the integration of various inflammatory and metabolic signals under physiological or pathological conditions. The initial paradigm for gasdermin activation was focused on protease processing and plasma membrane localization. An expansion of this paradigm witnesses the discovery of cleavage-independent pyroptotic activities for several mammalian gasdermins, mostly attributable to the different post-translational modifications. This echoes cleavage-independent activation of atypical gasdermins from ancient eukaryotes reported recently [107], which involves either release of disulfide-linked autoinhibition through conserved antioxidant system, or heterodimerization-triggered conformational changes and pore assembly.

Perhaps not surprisingly, it is now evident that these lipid-binding proteins may indeed associate with other organelle membranes as well. The organelle targeting instead of direct cytolysis may be a mechanism to amplify inflammatory signaling prior to cell death, especially for mitochondria targeting that in turn promotes gasdermin pore formation in a positive feedback loop. A major knowledge gap is the mechanisms for gasdermin targeting to different membrane compartments. What is known currently is that gasdermins harbor intrinsic affinity for negatively charged phospholipids, such as cardiolipin found almost exclusively in the inner mitochondrial membrane [4,61,62,67,89–91]. It remains to be determined whether the recently discovered gasdermin S-palmitoylation/acylation [79–83], which facilitates their association with plasma membrane, may also play a role in regulating their translocation at other organelle membranes. In addition, chaperone proteins and other cellular factors have been suggested to play roles in directing some gasdermins to different membranes [98,108]. How organelle-specific targeting may be achieved through a combination of intrinsic lipid affinity, post-translational modifications including lipidation, and utilization of chaperone proteins or other factors for different gasdermins will be an exciting area of investigation. The physiological context of such organelle targeting such as different cell types, tissues, metabolic status, and stimulation conditions will be of great interest. Whether gasdermins assemble pores at different organelles, and if so, whether these are distinct from those at the plasma membrane in terms of their architectures and functions is another intriguing area of study.

The diverse mechanisms of gasdermin activation and regulation in response to different upstream signaling pathways demonstrate the versatility of this conserved family of pore-forming proteins in various aspects of cellular physiology throughout evolution. The pleiotropic functions of gasdermins in inflammatory disorders, antimicrobial defense, antitumor immunity, neurodegenerative disorders etc., suggest fertile ground for exploration of therapeutic avenues. The many aspects of the pyroptotic and non-pyroptotic functions of gasdermins, such as protease cleavage, post-translational modifications, lipid binding and membrane association, and regulation of gene expression are potential opportunities for the development of direct and selective inhibitors or activators. Recent reports of small molecule inhibitors [109–113] and a selective agonist [84] for GSDMD have already provided a glimpse of the potential for future pharmacologic targeting of this and other gasdermins in various pathophysiological conditions.

## Perspectives

- Gasdermins are effectors for pyroptosis, with their NTDs bind membrane phospholipids and assemble oligomeric pores, whereas their CTDs are regulatory domains that play important roles in resting and activated states.
- The initial model for gasdermin activation is modified based on evidence of cleavage-independent pyroptotic activities of gasdermins upon post-translational modifications, as well as gasdermin localization at different membrane compartments beyond plasma membrane.
- Future studies will address a major knowledge gap regarding mechanisms for gasdermin targeting to different membrane compartments, which may be achieved through a combination of intrinsic lipid affinity, post-translational modifications, or utilization of chaperone proteins and other factors. The pleiotropic functions of gasdermins in inflammatory disorders, antimicrobial defense, antitumor immunity, neurodegenerative disorders etc., suggest fertile ground for exploration of therapeutic avenues.

## Open Access

Open access for this article is provided via our Subscribe to Open programme, made possible through the support of our subscribing institutions. Learn more here: https://portlandpress.com/pages/open_access_options_and_prices#conditional.

## Abbreviations

α-KG: α-ketoglutarate
ALS: amyotrophic lateral sclerosis
CTD: C-terminal domain
DFNA5: deafness, autosomal dominant 5
DMB: 6,7-dichloro-2-methylsulfonyl-3-N-tert-butylaminoquinoxaline
ELANE: Elastase neutrophil expressed
GI: gastrointestinal
GSDM: gasdermin
MLZE: melanoma derived leucine zipper extra nuclear factor
NTD: N-terminal domain
PARP: poly (ADP-ribose) polymerase
PDAC: pancreatic ductal adenocarcinoma
PJVK: Pejvakin
*Spe*B: *Streptococcus pyogenes* exotoxin B
TME: tumor microenvironment
